# Application of NSGA-II to Obtain the Charging Current-Time Tradeoff Curve in Battery Based Underwater Wireless Sensor Nodes

**DOI:** 10.3390/s21165324

**Published:** 2021-08-06

**Authors:** Daniel Rodríguez García, Juan-A. Montiel-Nelson, Tomás Bautista, Javier Sosa

**Affiliations:** Institute for Applied Microelectronics (IUMA), University of Las Palmas de Gran Canaria, 35015 Las Palmas de Gran Canaria, Spain; drgarcia@iuma.ulpgc.es (D.R.G.); montiel@iuma.ulpgc.es (J.-A.M.-N.); tomas.bautista@ulpgc.es (T.B.)

**Keywords:** underwater wireless sensor node, wireless power transfer, NSGA-II, design optimization

## Abstract

In this paper, a novel application of the Nondominated Sorting Genetic Algorithm II (NSGA II) is presented for obtaining the charging current–time tradeoff curve in battery based underwater wireless sensor nodes. The selection of the optimal charging current and times is a common optimization problem. A high charging current ensures a fast charging time. However, it increases the maximum power consumption and also the cost and complexity of the power supply sources. This research studies the tradeoff curve between charging currents and times in detail. The design exploration methodology is based on a two nested loop search strategy. The external loop determines the optimal design solutions which fulfill the designers’ requirements using parameters like the sensor node measurement period, power consumption, and battery voltages. The inner loop executes a local search within working ranges using an evolutionary multi-objective strategy. The experiments proposed are used to obtain the charging current–time tradeoff curve and to exhibit the accuracy of the optimal design solutions. The exploration methodology presented is compared with a bisection search strategy. From the results, it can be concluded that our approach is at least four times better in terms of computational effort than a bisection search strategy. In terms of power consumption, the presented methodology reduced the required power at least 3.3 dB in worst case scenarios tested.

## 1. Introduction

From the energy consumption point of view, an underwater wireless sensor node based on batteries or batteryless is divided in two subsystems. One is the energy consumer and the other is the energy provider [1]. The energy provider is a simple battery. Then, the sensor node has a predefined life time. Nevertheless, long term deployments of wireless sensor nodes’ applications require a wireless power transfer (WPT) system. The usage of a batteryless system or a rechargeable energy storage device determines the working mode of the WPT system [2,3]. In all the cases, the wireless sensor receives the required energy from the WPT system [4]. The great advantage of the usage of an energy storage device over a batteryless system is that the sensor node energy consumption may be greater than the maximum energy transmission allowed by the WPT system.

For instance, in the case of batteryless approaches presented in [5,6], the energy is provided by a WPT system to a local energy accumulator and then the measurement is executed. There also exists another common batteryless approach which does not include an energy accumulator [7]. Therefore, WPT provides the energy throughout the whole measurement.

Nowadays, the energy/power consumption optimization is a hot topic in the wireless sensor network research area. In the literature, there are several approaches at all design levels. At the highest level, the authors propose routing algorithms to minimize the energy consumption of the communications from a source and to a destination sensor node using other intermediate nodes [8]. Another high level optimization example is presented in [9], where the authors modify the digital high level communication layer to reduce the sensor node energy consumption during the usage of its transmission unit.

At the physical level, the wireless energy transmission adaptation is also studied in detail. This is the case of the study of multiple input and/or usage of output antennas in the energy transmission and reception sides of the sensor network. A multiple input and single output (MISO) system has been proposed like in [10], where the energy provider is composed of an array of antennas and the sensor node includes a single antenna. In terms of WPT for underwater sensor networks, the high signals’ attenuation at high frequencies promotes low frequency WPT solutions [11,12].

In the literature, most of the underwater sensor networks presents a WPT approach for autonomous underwater vehicles (AUVs). The mobility feature of the sensor nodes allows for using a docking or near field coupling solution where the vehicle is physically placed near the energy source. In terms of distance, these approaches cover only several dozen centimeters [13].

On the other hand, in the literature, the problem related to the WPT distance is solved using resonant coils or mixed wired-wireless solutions. The resonant approach consists of disposing several antennas working at a defined frequency [14]. These researchers applied the near field concept of the magnetic coupling which requires great dimension coils. The mixed wired-wireless solution is based on the same principle of the domino power transfer technique, but it inserts wire sections to extend the covering range at the cost of reducing the wireless charging area [15].

All of the approaches presented study in detail the sensor network from a circuit point of view, its power adaptation and finally demonstrate the working conditions to maximize the power transfer. However, all the solutions in the literature assume that the energy source does not have constraints in terms of energy. For example, in [14], the authors’ solution provides a maximum efficiency of 25% for a WPT in the range of 1 W to 100 W, up to a depth of 10 m using a high conductivity antenna with only 38.1 mΩ. In [15], the authors study the effect of the parasitic resistances of the antennas and determine that it is necessary to reduce those values to the minimum. The wired-wireless approach provides energy to up to five sensor nodes with −3.11 dB of power difference between sensor nodes using a 23 AWG standard for antennas and cables (620.16 mΩ).

Independently of the final application of the underwater wireless sensor network, in the literature, the energy requirements of the power source are not considered at all. From a practical point of view, its implementation despite the research advances is not possible, or at least it reduces its deployment considerably in most of the cases. For example, authors in [16] present a three-phase WPT system with a DC–DC 92.41% of efficiency. These results are based on Finite Element Method (FEM) simulations, and the circuit modeling also does not include the parasitic resistances of the coils. Moreover, this approach requires a current of 10 A and 100 V from its power source circuits.

The power requirements for the energy source of the practical underwater wireless sensor network implementation make the usage of an ocean surface solution like vessels, buoys or similar approaches mandatory [17,18]. At the ocean surface level, the energy source uses traditional power sources like a diesel engine-generator, or a renewable source generator like solar panels or wind turbines, among others.

In summary, the minimization of the required power from the energy source point of view is reached in the literature indirectly through the maximization of the energy transmission efficiency [19]. In addition, some other authors reduce the antenna parasitic resistances and minimize the generator equivalent impedance [20]. Moreover, the batteryless approaches in the literature also control the amount of energy transmitted to the sensor node turning on and off the energy source [21].

Recently, authors in [22] introduce a methodology that provides energy to a sensor network just when the sensor node must execute a job using the Radio Frequency IDentification RFID ISO 11784/11785 standard. The amount of energy transmitted to the sensor node is controlled turning on and off not only the energy source, since the sensor node self disconnection also contributes to reducing the required energy at the power source.

In this paper, we use a multi-objective optimization engine based on NSGA-II for minimizing the required energy from the power source point of view. The key contributions of this paper are:

- A novel design exploration based on the NSGA-II optimization engine is proposed to obtain the optimal Pareto front curve in terms of charging current–time of an underwater sensor node power source.
- In addition to approaches found in the literature, the amplitude of the signal used in the power transmission is included as a design parameter in the optimization.
- The proposed solution does not require the modification of any of the sensor networks’ elements or sensor nodes’ WPT parameters. In other words, the  required improvements of the proposed approach are only applied to its power source circuits.

The rest of this paper is organized as follows. In the next section, an overview of our proposed design exploration methodology is introduced. Then, Section 3 presents basic concepts about an underwater wireless sensor node, its energy wireless transfer, and battery modeling from a practical perspective. Our local design space exploration in NSGA-II is explained in detail in Section 4. Then, the design exploration is explained using several examples defining the rules to obtain the Pareto frontiers of the proposed application. The results are shown in Section 6, which also includes the comparison of our approach with literature solutions and also a bisection search strategy. This paper finishes with the most important conclusions of our research.

## 2. Design Exploration Overview

**Figure 1:**
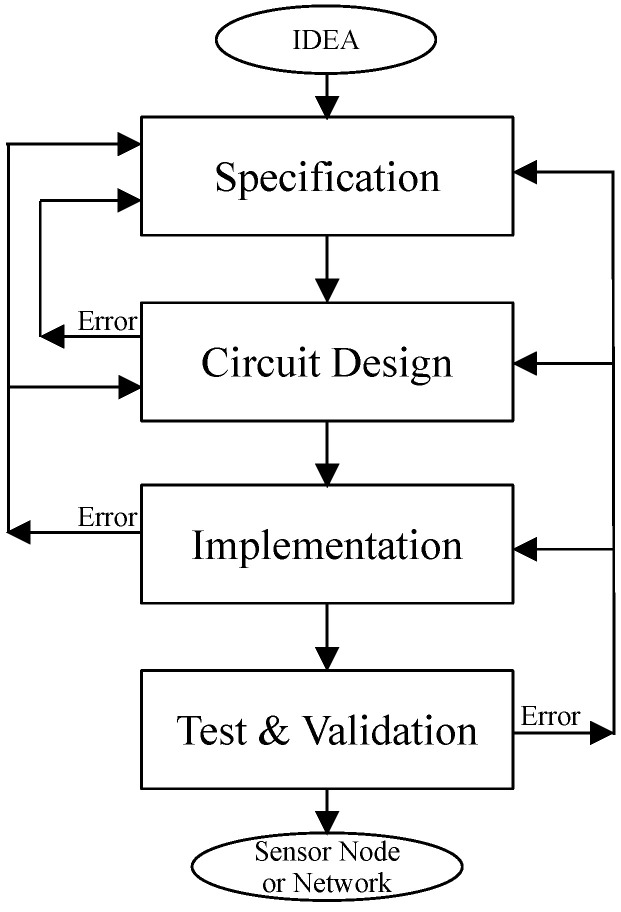
Traditional electronic design flow methodology [24].

It is well known that the design flow followed to implement a sensor network or node [23] is basically an electronic circuit design methodology [24]. It is not a straightforward task, and it is called design flow cycle. Figure 1 presents a simplified version of the traditional electronic design flow. Finishing the specification of the project idea, its circuits are designed. Then, the proposal is implemented, tested, and validated.

In general, each stage of the design flow includes a potential loop to the previous ones. The loops are executed when an error arises or when some refinement is required. These fatal events in the last stages produce a delay issue on time to market. This also requires a great fixing effort in the face of similar error in the initial stages.

The decisions made in the early stages of the design flow have consequences in later ones, and therefore on the final prototype. In this scenario, each set of design values derived from the design flow defines a unique design implementation X. The set of all possible design implementations is called Design Space D:

(1) $$D=\left\{X_{0},\cdots ,X_{i}\right\},\;\;\;\forall i\in N↔\exists X_{i}$$

The problem appears when two or more design variables are contradictory—for example, the antenna size versus the transmission distance, or the sensor node computational effort against the energy required. In other words, to obtain an optimized implementation makes it mandatory to minimize those design variables. However, the step-down of one variable implies the increasing of the other and vice versa.

From the designer’s point of view, a key idea is to obtain a closed formula that allows for determining these design parameters, such as for maximum energy transfer [20]. However, the increasing complexity of the sensor network or node also increases the complexity of its formulation to an unmanageable and incomprehensible level.

When it is not possible to obtain a formulation, the designer uses high level descriptions of their approaches and defines a set of values for those critical design parameters in order to evaluate their influence on the behavior of the design based on simulation [24].

In general, the value selection for those critical design parameters is made based on the designer’s know-how. The know-how based solutions $D_{nh}$ represent a subset of the design space ($D_{nh}$ ⊂ D). In other words, the number of implementations in the design space is huge in comparison with the know-how based solution subset:

(2) $$\left(\mathrm{sizeof}\left(D\right)=k↔\exists X_{k-1}\wedge ∄X_{k}\right)\wedge \left(\mathrm{sizeof}\left(D_{nh}\right)=h↔\exists X_{h-1}\wedge ∄X_{h}\right)\to k⋙h$$

Although the know-how implementations meet the design specifications, these solutions could be far from local/global optimal implementation of the design space. In this scenario, we define the design tradeoff curve TC as the set of optimal design implementations defined by at least two or more contradictory design variables.

Given a *fitness* function assuming its minimization, the TC is defined as:

(3) $$\mathrm{TC}\left(D\right)=\left\{X_{0},\cdots ,X_{j}\right\},\;\;\forall \;j\in N\;\wedge \;X_{j}\in D↔\exists \;min\left(fitness\left(X_{j}\right)\right)$$

As the design variables are contradictory between them, its fitness function has more than one minimum. The subset of design points that need those minima are the Pareto front of the design space D, which is the tradeoff curve TC. In this scenario, the D exploration and TC discover are not trivial tasks. However, in order to reduce the complexity of the problem, the search strategy could be performed following a divide and conquer strategy based on several local searches instead of a global one. In this case, the local design space LD can be defined as:

(4) $$D=\overset{k-1}{\underset{i=0}{⋃}}{\mathrm{LD}}_{i},\;\;\forall \;i,j\in \left[0,k-1\right]\wedge \;i\neq j\to \left({\mathrm{LD}}_{i}\cap {\mathrm{LD}}_{j}\right)=\emptyset$$

and, therefore, we can define the local tradeoff curve LTC as:

(5) $$\mathrm{TC}\left(D\right)=\mathrm{TC}\left(\overset{k-1}{\underset{i=0}{⋃}}{\mathrm{LD}}_{i}\right)=\overset{k-1}{\underset{i=0}{⋃}}{\mathrm{LTC}}_{i},\;\;\forall \;i\in \left[0,k-1\right]$$

where ${\mathrm{LTC}}_{i}$ is the local tradeoff curve of the local design space ${\mathrm{LD}}_{i}$.

### 2.1. Target Application

As presented above, our target in this research is to develop a design exploration methodology to minimize the energy transmission from the perspective of the source of the power supply of an underwater wireless sensor node/network. In order to understand our approach, it is better to know the target application and working scenario first.

Assuming that the power supply of a sensor network or node is a finite energy source, their specifications as voltage or current power source could be crucial in applications nowadays. For example, from a sensor network design point of view, a power source based on a diesel engine-generator can be considered at first sight an unrestricted power source. However, from an industrial deployment point of view, the diesel consumption is a very important design variable. In most cases, this design variable defines the economic viability of the application, e.g., in aquaculture industry, the tanks, and offshore fish farm cages.

The optimal solution from an industrial point of view is the use of renewable energy, that is, to use some energy harvesting approach based on solar, wind, or kinetic energy, among others. Certainly, this alternative solution introduces more restrictions in terms of available energy at the power supply than the solution using a diesel engine-generator.

**Figure 2:**
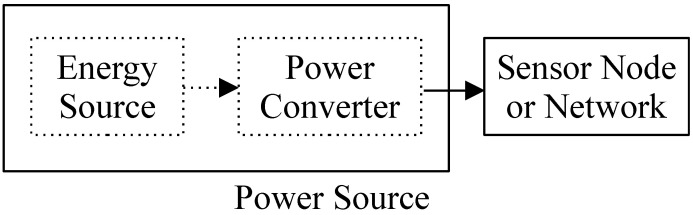
Simplified block diagram of a sensor network from the power source point of view.

Figure 2 presents a simplified block diagram of a sensor network from the power source point of view. There are mainly two blocks: the sensor network (energy consumer) and its power supply (energy provider). Nowadays, the sensor network is designed to minimize its energy requirements as it was appointed in the Introduction section. On the other hand, the power source must provide the energy needed. In general, a sensor network power source is composed of two basic subsystems: an energy source and a power converter. The power converter is basically a direct current to direct current (DC-to-DC) converter or a DC to alternate current (DC-to-AC) converter.

The research area of DC-to-DC/AC converters is highly mature. There is a wide range of approaches in the literature with multiple features like high power efficiency and controllable output voltage for variable load conditions among others [25]. Since the energy source is finite, it is possible to model its behavior as a battery with the right values like, for example, the maximum current, the nominal voltage, and the charge or remaining energy, among others.

In the case of a power source based on energy harvesting, the harvesting effect is modeled by increasing the remaining energy with time. On the other hand, the complexity of nowadays sensor networks allows for executing different experiments, e.g., turbidity and waves frequency, among others. Those experiments/measurements define several power consumption profiles which correspond to their particular energy needs. Fitting the energy offered by the power source to the required by the sensor network or node is the objective of this work.

### 2.2. Proposed Methodology

**Figure 3:**
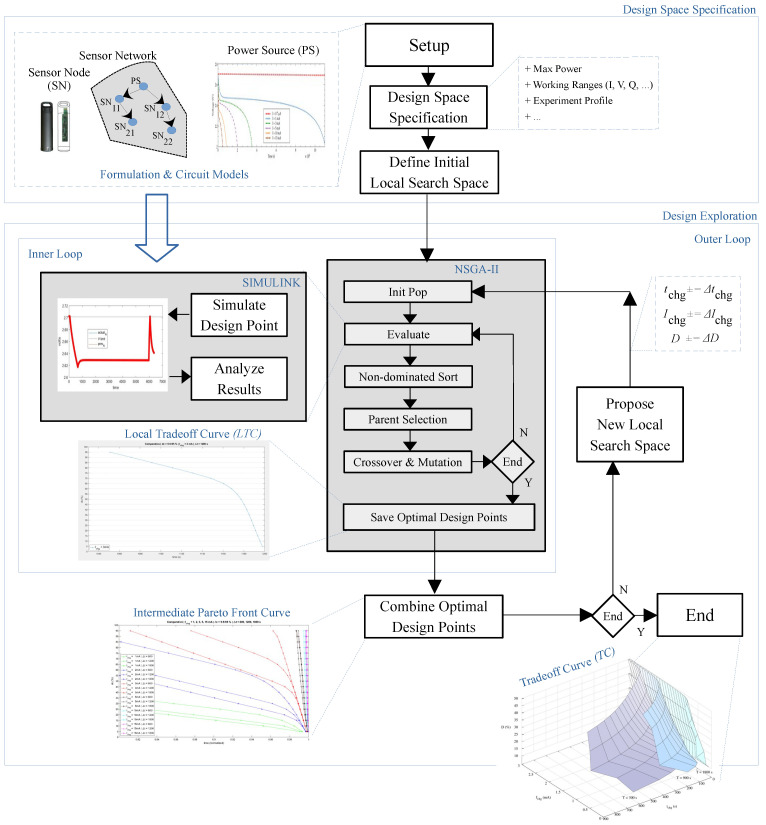
Proposed design space exploration methodology.

Figure 3 presents the block diagram of our proposal from a high level point of view. Our methodology requires the formulation and/or circuit models related to the used sensor node, network, and power source. Then, the specifications of the design space must be provided, which include the working ranges of the design variables and network execution profile, among others. Before starting the search, it is mandatory to define an initial working range to guide the first search.

In general, this initialization is set to the complete design space D. However, this step allows for guiding the exploration in whatever specific and desired local design space range. In this manner, the methodology can be used to refine some previously computed tradeoff curve or just to focus the search on the designers’ implementation areas of interest.

Our proposal implements a double nested loop. The inner one obtains the local tradeoff curve LTC between the required charging current and time for the working ranges. The outer loop combines this information for obtaining the Pareto front curve.

Our inner loop is basically an implementation in Matlab of the Nondominated Sorting Genetic Algorithm II (NSGA-II). The proposal follows its original execution scheme, that is, after its initialization, NSGA-II executes a loop with the evaluation, shorting, tournament, and finally the crossover and mutation of its population.

The key point explained in a few words is that the NSGA-II population represents a set of design implementations, one X per individual. For this reason, the evaluation stage of the NSGA-II requires using the Simulink circuit simulator. From each simulation, the inner loop analyzes the results and obtains the required feedback, e.g., voltages, currents, charge and execution parameters, among others.With these data, the fitness function can then be computed.

The population of each NSGA-II generation defines a set of design implementations candidates to be part of the tradeoff curve TC:

(6) $$Pop=\{X_{0},\;\cdots ,\;X_{i}\},\;\;i=\mathrm{Population}\mathrm{size}-1$$

Once the NSGA-II reaches the last generation, the best design points build the so-called local tradeoff curve LTC.

On the other hand, the outer loop modifies the working ranges based on the design parameters of the inner loop exploration to maximize the searching coverage over the complete design space. In other words, the outer loop guides the local search performed with the NSGA-II exploring the design space and obtaining its Pareto frontier.

## 3. Practical Application

Nowadays, the complexity of practical underwater wireless sensor nodes increases dramatically with the number of features included on board. For example, each sensor device requires its own hardware, setup, and/or calibration. In addition, some measurements make a pre-processing stage mandatory. Finally, the data obtained are stored locally in a flash memory or sent to a data server through its communication unit. On the other hand, the battery or energy accumulator of the wireless sensor node of the WPT system is modeled to guarantee the energy for executing the programmed task/job. This programmed task/job is usually a measurement.

In order to demonstrate the practical application of our approach to a real wireless sensor node, we use the underwater wireless sensor node presented in [15] as a target of the system under optimization.

### 3.1. Underwater Wireless Sensor Node

**Figure 4:**
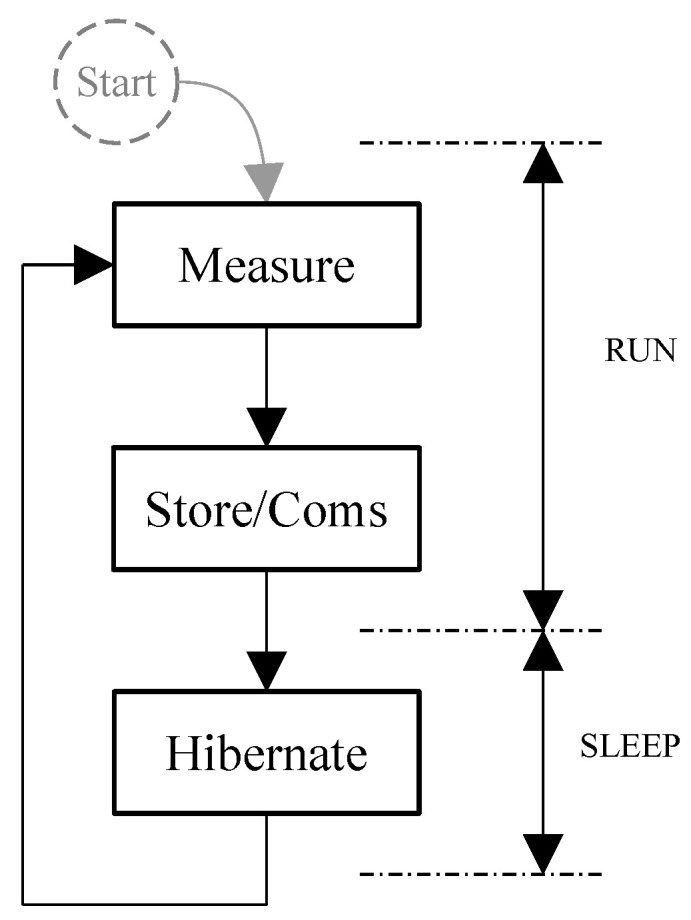
Simplified high level block diagram of the behavior of the sensor node.

Despite the exposed complexity, the main purpose of this kind of sensor node does not differ from traditional ones, which is to measure one or several physical variables. Depending on the sensor node capabilities, the acquired data are saved locally or transmitted to a remote memory. Figure 4 presents a simplified high level block diagram of the behavior of the sensor node implemented.

In general, the sensor node executes a single measurement. However, most of their applications requires monitoring of a single or several physical variables. This involves repeating the measurement in a timed manner. For example, the sensor node executes a set of measurements ruled by a scheduler. In other words, it executes iteratively the measurement and data storage or transmission in a time controlled loop. At the last step, the sensor node hibernates to save energy between iterations.

Despite of the simplicity of the block diagram presented in Figure 4, there are great differences in terms of power consumption between the measure, store, communications, and hibernation steps. In addition, the measurement step performs in most of the cases’ different tasks. Therefore, in terms of energy optimization, the complexity of the scheduler must be taken into consideration, i.e., hibernation times and measurement types. This implies that this straightforward block diagram produces a sequence of non-equal energy demands per iteration. In this research, we call execution profile to this timed sequence of energy demand. Finally, each different measurement schedule produces a unique execution profile.

### 3.2. Wireless Power Transfer

**Figure 5:**
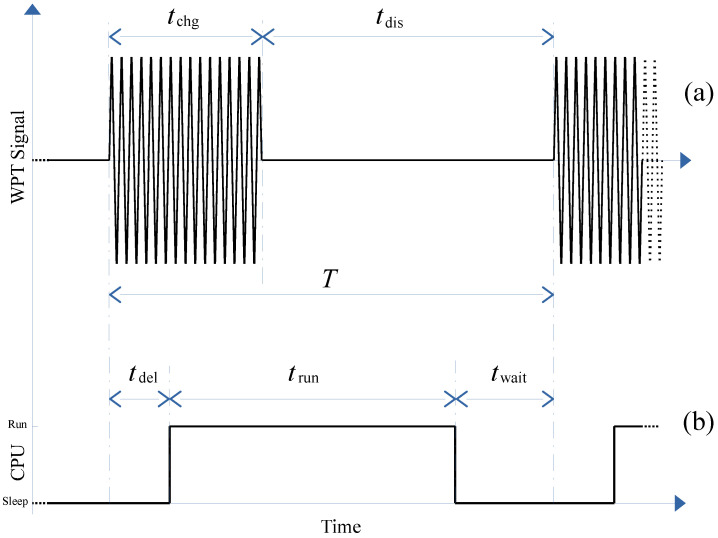
Sensor node using the ISO 11784/11785 standard: (**a**) wireless charging transfer signal and (**b**) underwater sensor node working mode.

In this research, we use the underwater sensor node presented in [22] as a reference. This sensor node follows the ISO 11784/11785 standard. This RFID standard defines both the WPT and data transmission protocols. The research of this paper is focused on the WPT usage. The data transmission remains unaltered and functional. Figure 5 depicts a complete measurement iteration. The upper curve represents the WPT signal. Figure 5b shows the sensor node working modes.

Given an iteration time *T*, the sensor node receives energy during the first $t_{chg}$ seconds (see Figure 5a). Then, the WPT signal disappears during $t_{dis}$. In the sensor node side, once the iteration begins and the WPT signal is applied, the CPU of the sensor node requires some delay ($t_{del}$) to go from the sleep mode to its run one. Then, the programmed measurement is executed during $t_{run}$. Finally, the sensor node goes into hibernation waiting for the next iteration.

From Figure 5, we may formulate:

(7) $$T=t_{chg}+t_{dis},$$

and

(8) $$T=t_{del}+t_{run}+t_{wait}$$

In several practical applications, the WPT charging signal is not present during the execution time $t_{run}$ because it generates an unacceptable noise in the measurement devices, causing an error. This scenario defines a worst corner case. A fast and simple solution is to set $t_{del}$ equal to or greater than $t_{chg}$ to avoid this problem. In this research, we set $t_{del}=t_{chg}$. As a consequence, in addition to Equations (7) and (8), now we define:

(9) $$t_{dis}=t_{run}+t_{wait}$$

### 3.3. Battery Model

The battery model is the other important key issue. The mission of the battery is to store and provide the energy to the sensor node. Its charging and discharging behavior has a great impact on the design parameters like the system life time and available energy, among others. In our research, the ultra low power design feature implies to supply the minimum energy-time product required to ensure the right execution of the measurements. The charge and discharge behavior of a battery is not a linear function. It follows the Equations (10) and (11) for Li-on batteries [26] like the ones used in this research.

(10) $$V_{chg}=E_{0}-R_{int}\times i-K_{c}^{\prime }\times \frac{Q}{it-0.1Q}\times i^{*}-K_{c}^{\prime \prime }\times \left(\frac{Q}{Q-it}\right)\times it+A_{c}\times e^{-B_{c}\times it}$$

(11) $$V_{dis}=E_{0}-R_{int}\times i-K_{d}^{\prime }\times \frac{Q}{it-Q}\times i^{*}-K_{d}^{\prime \prime }\times \left(\frac{Q}{Q-it}\right)\times it+A_{d}\times e^{-B_{d}\times it}$$

where $E_{0}$ is the called thermodynamics voltage (V), and $R_{int}$ represents the internal resistance (Ω). *Q* defines the battery capacity (Ah). The polarization constants are labeled as *K* and basically they are fitting values (V/(Ah)). In addition, *A* coefficients define the exponential zone amplitude (V) and *B* coefficients specify the exponential time constant inverse value (1/(Ah)). Note that, while *i* is the battery current, $i^{*}$ represents *i* filtered by a low pass filter, with a cutoff frequency of $1/T_{d}$ and $T_{d}$ is the battery response time (s). Finally, it is the actual battery charge (Ah).

**Figure 6:**
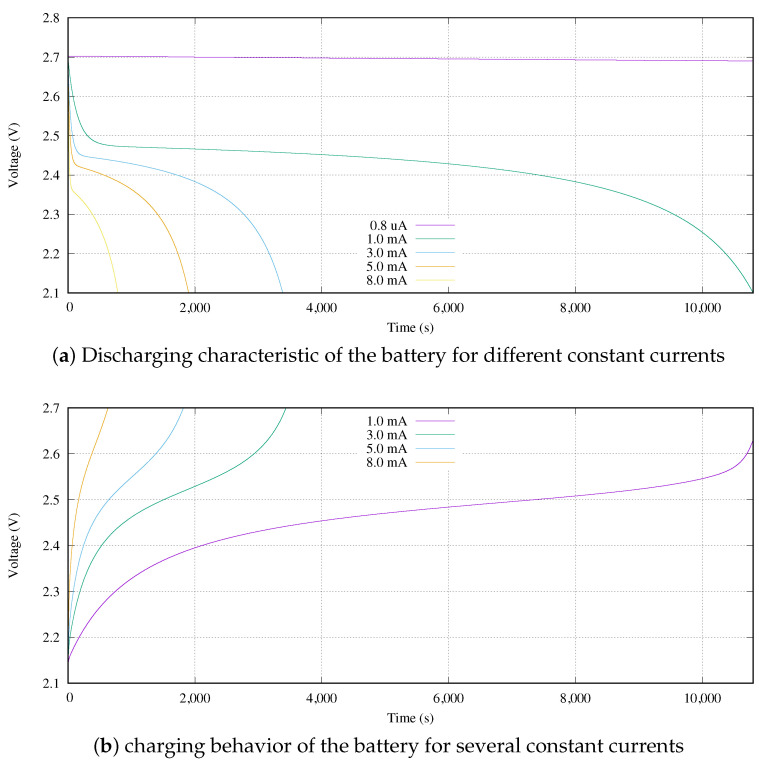
Voltage behavior of a 3 mAh battery.

From another perspective, Figure 6 shows this behavior based on simulations. These curves represent the output voltage of the battery given constant discharge ($I_{dis}$) and charge ($I_{chg}$) currents. Both behaviors are divided into three working areas. In case of the discharge, see Figure 6a, the first one corresponds to an exponential function. In terms of time, it is located at the start of the discharging process, and it represents a short time period in comparison with the other areas. Then, a quasi-stable voltage area occurs which corresponds to its nominal value area. Finally, once the battery voltage reaches its nominal voltage value, the battery is considered close to its full discharge and an exponential behavior happens again.

Figure 6b shows the charging feature of the Li-on battery modeled by Equation (11). The working areas of the charging process are the same as for the discharging process, but they are disposed in reverse order. First, there is an exponential behavior due to a low energy storage level that is the discharging area. Then, a semi-linear voltage increasing occurs during a long time (nominal area) in comparison with other areas. Finally, the voltage of the battery grows exponentially. Moreover, the discharging and charging curves are similar in behavior, although they are not equal.

From a practical point of view, in this research, we use the battery model UMAC from Murata Manufacturing Co. Ltd. (Nagaokakyo, Japan). Its main features are detailed in Table 1. We need to take into consideration that, although the usable battery working range is [1.8, 2.7] V, it can be used outside of its margins. However, the 1.8 V voltage can be only reached when the battery is under a specific load. Otherwise, its output voltage is greater than 1.8 V. For example, given a battery at a temperature of 25 ºC, its output voltage is 2.1 V when it is without load (zero current load).

In addition, the battery may be charged to a voltage greater than its upper limit. In our case, we never break this limit. Finally, a high current might be used for charging the device (fast charging). For this battery, an $I_{chg}$ higher than 9 mA is considered a high current. However, the battery degradation effect is greater with greater charging currents. The battery manufacturer indicates that it is mandatory to perform a non-high current every three or four fast charging cycles.

## 4. Local Design Space Exploration

As was pointed out in the previous Section 2.2, the inner loop executes the local search strategy based on an NSGA-II optimization engine. Although it is a well known heuristic procedure, it is mandatory to evaluate its working conditions and results’ usefulness in each new application. In the following sections, we first expose some practical considerations about the application example used in this paper. After this, the problem codification and fitness function are taken into consideration. Another important issue is to determine the convergence of this heuristic approach. At the end of this section, the criteria for considering a design point optimal and that it belongs to the tradeoff curve TC are presented.

### 4.1. Practical Considerations

Once the WPT system activates its charging signal, from the sensor node point of view, it is desirable to reach exactly 2.7 V as target charged voltage. However, we need to take into consideration that looking at Figure 6a, there is not so much difference in terms of stored charge between 2.7 V and 2.6 V. This is because those voltages are located in the exponential behavior of the battery. The stored charge varies only 0.7% between two equal batteries with those two voltages when $I_{dis}$ is equal to 1 mA. In case of a discharge current of 3 mA, this percentage is only 0.5%.

In this sense, we determine that the solutions provided by the NSGA-II optimization engine are candidates to be in the Pareto front if the obtained charged voltage is 2.65 V < $V_{chg}$ < 2.75 V. In this research, we named these voltage margins as target voltage range. Otherwise, those solutions are discarded from the design space exploration at high level by the Design Exploration procedure (see Figure 3).

### 4.2. NSGA-II Codification and Fitness Functions

In our approach, an individual represents a circuit realization. Each implementation is defined by a charging current $I_{chg}$ and time $t_{chg}$. For this reason, we define an individual as an array of two chromosomes defined with real values. In order to ensure the practical implementation of the proposed circuit setup, it is mandatory to define the limits of each chromosome before starting the search.

Then, a population is a set of different practical circuits implementations, corresponding to a circuit per individual. The evolution of the population along the generations produces the refinement of the circuits’ features.

The fitness function must help to the NSGA-II optimization engine to find the set of optimal circuit parameters $I_{chg}$ and $t_{chg}$. As it was pointed out in previous Section 4.1, an important key is that the battery is ready to be used if its voltage is within a target voltage range. Therefore, we define the first fitness function as:

(12) $${Fitness}_{1}\left(Ind\right)=\left|V_{meas}-V_{target}\right|$$

Given the individual $Ind=\{I_{chg},t_{chg}\}$, $V_{meas}$ is the voltage obtained by circuit simulation at the end of the charging period $t_{chg}$ when its charging current is $I_{chg}$.

In addition, our approach formulates a second fitness function as:

(13) $${Fitness}_{2}\left(Ind\right)=t_{chg}\times I_{chg}\times \beta_{i\times t}$$

This last equation represents the weighted charged energy in the battery when $I_{chg}$ is applied during $t_{chg}$. Note that we included the $\beta_{i\times t}$ factor to scale the chromosomes variability due to the scale difference between charging currents in 10${}^{-3}$ amperes and charging times in seconds.

### 4.3. Convergence

**Figure 7:**
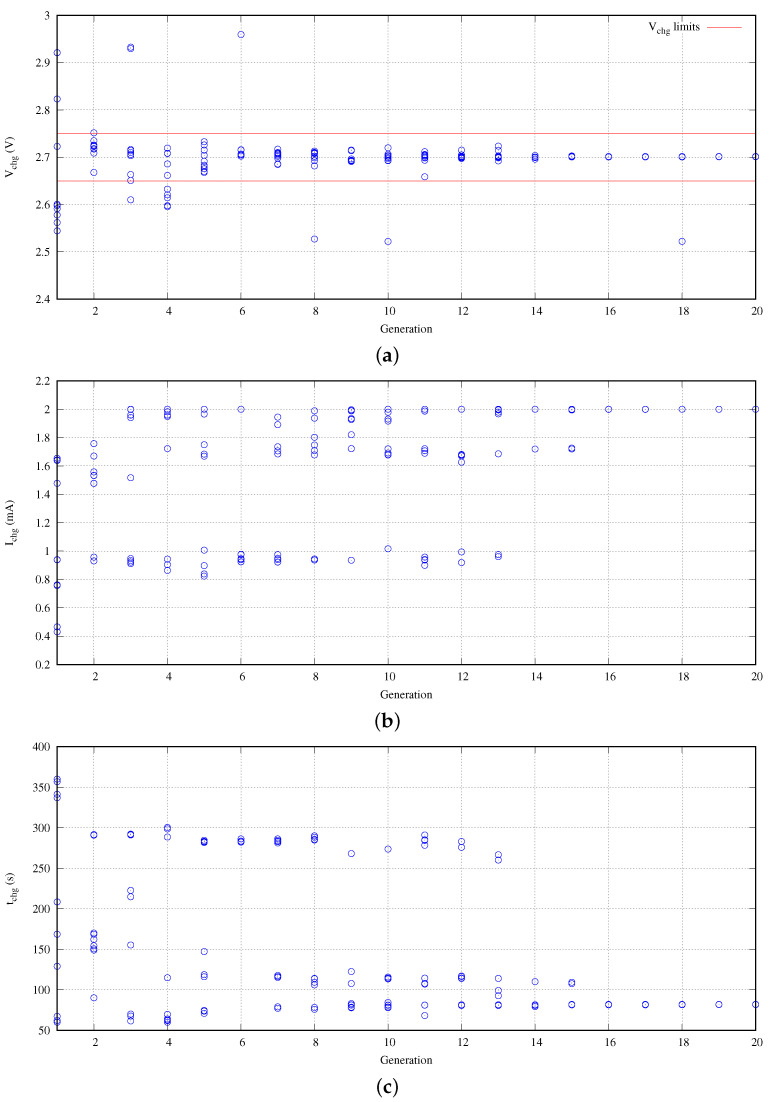
NSGA-II approach convergence: (**a**) obtained charging voltage, (**b**) required charging current, and (**c**) required charging time.

It is time to check the convergence of the solutions provided by the optimization engine. In this experiment, we are trying to discover the optimal design point within a defined working range. The population of the experiment is set to 10 individuals. The population is evaluated during 20 generations. As was pointed out previously, each individual represents a complete design with its own particular parameters. Figure 7 shows the typical evolution the NSGA-II solutions in our approach.

In this experiment, the search range of the design space is 0.2 mA $<I_{chg}<$ 2.2 mA and 300 s $<t_{chg}<$600 s. Figure 7a represents the convergence of the target voltage to charge $V_{chg}$. We conclude that our solution requires only seven generations obtaining an error lower than [−7.83, +34.6] mV. Obviously, the higher the number of generations, the greater the convergence. For example, after the fourteenth generation, the voltage deviation is lower than ±4 mV. Certainly, along the evolution, there are multiple individuals that do not meet the error criteria. However, they are necessary because they belong to the crossover and mutation capabilities introduced to avoid local optimum design points in the NSGA-II optimization engine. Despite of their presence at this search level, they are discarded at high level design explorations as was pointed out in practical considerations (Section 4.1).

Figure 7b presents the convergence of the required charging current. In terms of global behavior, the optimization engine found three clear optimal currents in the search. The first one corresponds with the maximum current available in the search space range, that is, 2.0 mA. The second one is close to 1.7 mA. Finally, the other optimal design point is close to 1 mA. However, these two last optimal points are discarded by the NSGA-II optimization engine after generations 13 and 15. After this non-return point, the obtained solutions belong to the maximum current.

This last effect is observed in Figure 7c from the point of view of the required current to reach the target voltage. As soon as the $I_{chg}$ is increased, the required time $t_{chg}$ is decreased. Finally, the current in terms of generations follows the same behavior than the required time in reverse form. Moreover, a mirroring effect between Figure 7b,c is produced.

### 4.4. Design Space Search

Once the problem convergence is guaranteed, other important key point is to determine the search coverage over the design space. Our main idea is to gain a high degree of confidence that the vast majority of optimal design points are found. Nevertheless, the heuristic nature of the optimization engine does not allow us to ensure that. However, the spatial distributions of the individuals along the design space provide an idea of the search coverage.

**Figure 8:**
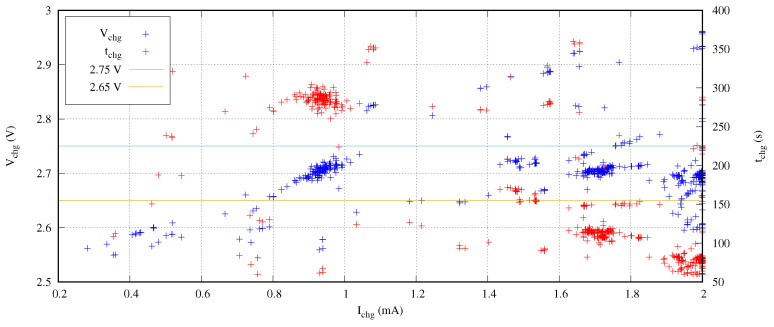
Search coverage of several searches with different seeds.

In order to evaluate the spatial distribution, we executed the previous experiment 15 times with different seeds. Figure 8 presents the results of this exploration in terms of target voltage versus the charging current and the required time. This figure includes all the design points evaluated by the NSGA-II optimization engine to our fitness function.

In this Figure 8, the candidates to be optimal design points are those solutions enclosed between the orange and the blue horizontal lines for the target voltage represented by blue points. We observe several grouped solutions that belong to local optimal design points. The other non-grouped solutions are the trials of the optimization engine searching in the design space.

From a circuital point of view, the increase of the charging current produces an increase of the target voltage. In a similar way, an increase of the charging time also increments the target voltage. Moreover, lower charging currents require higher charging times to reach a target voltage. This is the reason why the trials are concentrated under an imaginary diagonal line from the left-bottom to the right-top in this Figure 8. Under this imaginary diagonal line, the coverage distribution obtained throughout the entire search is evenly spaced.

### 4.5. Pareto Front Design Points

For a proper search space exploration, it is mandatory to discard the non-optimal design points. A quick and simple rule to select the candidates is to remove those solutions which do not belong to the target voltage range. An additional criterion is to short the solutions in terms of required charge.

**Figure 9:**
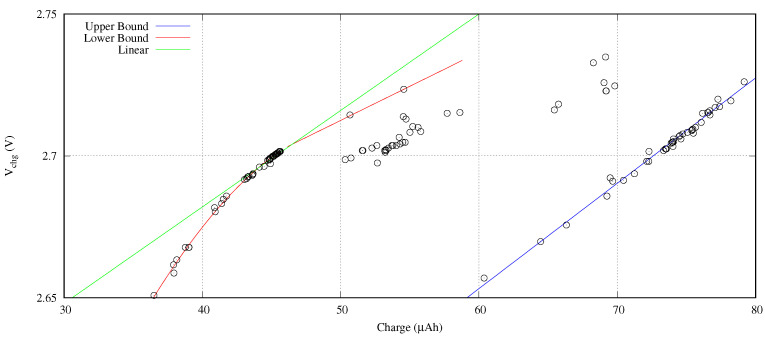
Target voltage against charge Pareto front curve.

Applied the first rule, Figure 9 presents the set of candidate solutions to be optimal design points obtained from the experiments presented in Section 4.3. In this figure, the horizontal axis shows the charge pushed in the battery, that is, $I_{chg}\times t_{chg}$. The vertical axis provides the reached target voltage. We clearly observe two frontiers: an upper bound highlighted with a red line and a lower bound remarked with a blue line. The red line defines the best solutions in terms of required charge. The upper bound is composed of those design points where the $I_{chg}$ is set at its upper limit (2 mA) of the design space search.

Despite the lineal function behavior depicted by the upper bound, the lower bound follows a quadratic law. The charge underestimation of the linear behavior (see green line in Figure 9) in comparison with its quadratic function is a 16.47% for minimum charge optimal design points ($I_{chg}\times t_{chg}$ < 36.2 ±0.1μAh).

## 5. Design Space Exploration

Based on the the reasoning shown in the previous section, we can determine the optimal current $I_{chg}$ and time $t_{chg}$ sets to guarantee the execution of the next measurement iteration *T*. However, in practical sensor node networks, this time *T* is not fixed, and it depends on the nature of the executed measurement (execution profile). Therefore, we need to consider the *T* parameter as a design variable to evaluate during the design space exploration.

Adding a new design variable to the problem requires the introduction of a new figure of merit (FoM) to evaluate the new optimal solutions as follows:

(14) $$\mathrm{FoM}=\frac{T-t_{run}-t_{chg}}{T}$$

This FoM assumes that $t_{run}$ reduces the available time to charge the sensor node. In other words, it defines the worst case scenario where it is not possible to charge and run at the same time.

Finally, we compare our approach with traditional methodologies where the sensor network supplies energy to the sensor nodes while they are not executing measurements. In this sense, we define the working duty cycle *D* as:

(15) $$D=\frac{t_{run}}{T}\times 100$$

After formulating this last equation and using all the previous information, we are able to implement an algorithm to explore and discover the tradeoff curve of a sensor network or node design space as shown in the following.

### Detailed Exploration Algorithm and Discussion

Algorithm 1 shows an implementation of our proposal described in pseudocode. As we advanced in Section 2.2 (see Figure 3), the design space exploration methodology requires the involved power source PS and sensor node/network SN models. As the search is based on simulation using the Simulink software from Mathworks, those models are specified with a black box using formulas or a schematic circuit description.

The design space D must be clearly defined. Therefore, in addition to the description of the PS and SN models, its limits DV must also be specified. Since D can be expressed as a grouping of several disjoined subsets ${\mathrm{LD}}_{i}$ (see Equation (4)), the DV is an array that contains a unique set of design variables ranges associated with each ${\mathrm{LD}}_{i}$. This means sizeof(DV)=sizeof(D)=k

Comparing this algorithm with the block diagram presented in Figure 3, the outer loop begins in its line no. 5 and continues up to line no. 30. On the other hand, the inner loop is located from line no. 11 up to line no. 24.

As we indicated previously, the inner loop follows the traditional NSGA-II algorithm. The key point in the inner loop is its evaluation stage (see lines from 15 to 18). This stage requires to simulate each individual X which represents a design implementation. The probed simulation variables provides the input data for the fitness functions.

Finished the inner loop, the next step is to extract the LTC from the NSGA-II population Pop. The last stage of the outer algorithm is to determine the next new search local space. Obviously, this is probably one of the most important issues in the design space exploration, taking into consideration that the local search is fully guided by the NSGA-II optimization engine.

This last issue has been widely studied and, for a long time, an unsolved problem in the electronics design automation EDA literature [24]. Although most of the studied cases are defined as NP-hard [27] problems, it is well known that it is possible to define an ad-hoc heuristic search strategy for a particular case where the design space has been previously completely studied. However, a minimal variation of a reduced set of their design variables or models makes the best solution in the literature fail.
**Algorithm 1** Design space exploration. 1: **procedure** Explore(PS,SN,DV) 2:     PS: Power source model 3:     SN: Sensor node/network model 4:     DV: Sets of design variables ranges ($DV=\left\{{\left\{{\bar{I}}_{chg},{\bar{V}}_{chg},\bar{E},{\bar{t}}_{chg},\bar{T}\right\}}_{i}\right\},\;\forall i\in$      [0,k−1],   ${\bar{I}}_{chg}=\left[min\left(I_{chg}\right),max\left(I_{chg}\right)\right],\;{\bar{V}}_{chg}=\left[min\left(V_{chg}\right),max\left(V_{chg}\right)\right],\;\bar{E}=$
      [min(E),max(E)],   ${\bar{t}}_{chg}=\left[min\left(t_{chg}\right),max\left(t_{chg}\right)\right],\;\bar{T}=\left[min\left(T\right),max\left(T\right)\right]$)

 5:     /* Outer Loop Start */ 6:     TC←∅;▹ Empty Tradeoff Curve 7:     iLS←0;▹ Init local search index 8:     k=sizeof(DV);▹ Number of local search subsets 9:     **repeat**10:         $LS\leftarrow DV_{iLS}$;▹ Get design space subset11:         /* Inner Loop Start */12:         Pop← initPopulation(PS,SN,LS);▹ Initialize first population13:         $Gen_{i}\leftarrow 0$;▹ Generation index to zero14:         **repeat**15:            **for all** X∈Pop**do**▹ Evaluate population16:                Simulate(PS,SN,X);▹ Call Simulink using the proposed▹ individual implementation17:                Evaluate($Fitness_{1}\left(X\right),Fitness_{2}\left(X\right)$);▹ Compute Equations (12) and (13)18:            **end for**19:            Sort(non-dominated, Pop);▹ Categorize proposals20:            Tournament(Pop);▹ Select parents21:            CrossAndMut(Pop);▹ Obtain new offspring22:            $Gen_{i}\leftarrow Gen_{i}+1$;▹ Increase generation23:         **until** $\left(Gen_{i}==Gens_{max}\right)$▹ Check last generation24:         /* Inner Loop End */25:         ${\mathrm{LTC}}_{iLS}\leftarrow$ ExtractLocalTradeoffCurve(Pop);▹ Save optimal design points26:         TC = $\mathrm{TC}\;⋃\;{\mathrm{LTC}}_{iLS}$;
27:         /* Propose next local search */28:         iLS←iLS+1;▹ Next, search space subset29:     **until** (iLS == *k*)▹ Check next outer iteration30:     /* Outer Loop End */     31:     SaveAndPlot(TC,FoM,D);▹ Present Pareto front curve
▹ using Equations (14) and (15)32: **end procedure**

In our approach, we propose a divide and conquer strategy for solving the local search of the tradeoff curve TC. We also propose to use the designers’ knowledge about their design for partitioning the design space D. The description of this methodology of global search is:

1. Determine design variables and its working ranges DV.
2. Execute the proposed Algorithm 1.
3. Determine uncovered areas in the TC obtained.
4. Partitioning the D and re-run the search algorithm.
5. Repeat the previous two steps until the uncovered areas are removed.

As each sensor network or node approach on the literature has its own design variables, the first step is to determine the design variables. Obviously, this apparently simple step requires good knowledge from the designer because a mistake in the design variable selection will lead to the failure of the methodology. For instance, the example of Section 4.3 defines as a design variable the charging current $I_{chg}$. In this scenario, the $V_{chg}$ is a variable depending on $I_{chg}$ because, in this example, we cannot control the load value. If we try to define both design parameters as design variables, in this example, the algorithm fails at the inner loop in the simulation stage.

The second step of this global search methodology is quite simple, since it is to execute the proposed algorithm over the complete design space. This provides the TC. However, in the worst case, some sections of the TC will be uncovered. This is the reason for the third step objective, which is to detect the TC uncovered areas. Once they are identified and located, the proposed algorithm is executed again for the design subsets with an uncovered area. Finally, the third and fourth steps are repeated until the uncovered areas are removed.

## 6. Results

In order to evaluate our methodology proposed for exploring and discovering the tradeoff curve, we selected a classical application measurement in aquaculture. This consists in measuring the turbidity [28] at the bottom level of an offshore fish farm cage like the one presented in [15]. This application measurement can also be applied to a tank based aquaculture solution like that presented in [29]. In addition, we use the batteryless and underwater sensor node presented in [22]. However, we modified its implementation to include the secondary UMAC battery from Murata, Ltd. as a capacitor of the sensor node (see Table 1). Finally, as design specification, the turbidity must be measured every 30, 15, and 5 min. This corresponds to 1800, 900, and 300 s, respectively.

### 6.1. Pareto Frontiers

**Figure 10:**
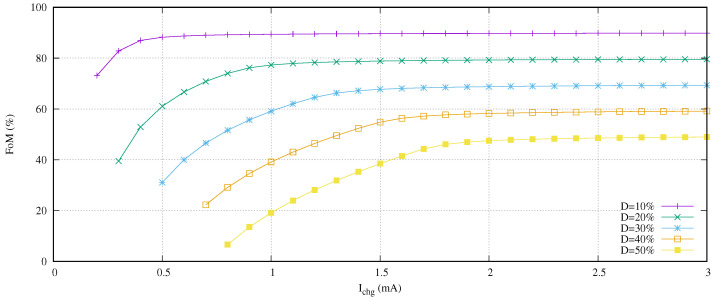
FoM, charging current and duty cycle behavior for a given experiment with T=900 s.

In this scenario, it is time to evaluate the design space behavior depending on the charging current and the duty cycle for a constant *T* for the given Equation (14). In this first experiment, we propose to move the duty cycle from a 10% up to a 50% in 10% steps for *T* equal to 900 s. In other words, the charging time ranges are [0, 810] and [0, 450] seconds for a 10 and 50% duty cycle, respectively. On the other hand, we set the charging current range as [0.1, 3.0] mA. Figure 10 presents the results of this experiment.

From a practical point of view, the proposed FoM measures the percentage of energy saved. For example, if the duty cycle is set to 10% and T equals to 900, this percentage means that $t_{run}$ is equal to 90 s and $t_{wait}+t_{del}$ is 810 s. If we use a hypothetical Dirac Delta function charger with $t_{chg}$ close to zero, the FoM (Dirac Delta function) will be 90%. Therefore, we can strictly ensure:

(16) FoM(%)<100−D(%)

This FoM upper limit is observed in the experiment data shown in Figure 10. For example, the 20% duty cycle curve is close to the 80% value after an $I_{chg}$ equal or greater than 1.5 mA.

On the other hand, it is shown that the definition of the curves do not fulfill the complete set of possible charging currents. For example, given that *D* equals to 30%, its minimum charging current is close to 0.5 mA. We must take into consideration that sometimes, although we are working with practical working ranges for $I_{chg}$ and $t_{chg}$, several combinations of them do not allow for charging the battery. Those limit points define the Pareto Front (PF) curve of the minimum charging current for a duty cycle $PF(\min\left(I_{chg}\right),\left(D\right))$. As we expect, its behavior shows that the increase of the duty cycle requires less current to charge the battery.

Moreover, we also observe in this Figure 10 that, at low charging currents, a small increment in its value produces a higher increase of FoM in comparison with high charging currents. This scenario defines a new PF curve. First of all, we need to define the terms’ high and low charging currents in order to obtain this curve.

In this research, we define the limit between high and low current as:

(17) $$I_{chg}^{*}=I_{chg}\;|\;|\mathrm{FoM}\left(\max\left(I_{chg}\right)\right)-\mathrm{FoM}\left(I_{chg}\right)|<5\%,\;\forall I_{chg}\in \left[\min\left(I_{chg}\right),\max\left(I_{chg}\right)\right]$$

**Figure 11:**
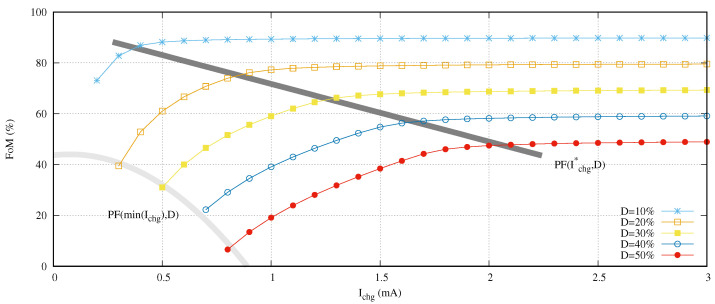
Pareto front curves for minimum charging current and high/low charging currents.

This equation defines the current at the point where the FoM decreases 5% in comparison with its full scale. Both Pareto front curves are shown in Figure 11. The high/low charging current limit is quasi-linear (|error| < 2.5%), and the minimum charging current follows a quadratic function.

**Figure 12:**
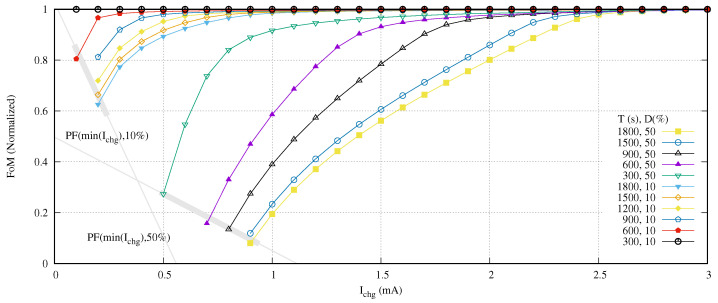
Normalized FoM for different duty cycles and *T*.

Exposing the behavior of the design space for a given *T*, it is time to modify this value and observe its influence in the FoM. Then, we propose as an experiment to compare the effect of duty cycles of 10% and 50% for different iteration times *T* from 300 to 1800 s in 300 s steps. Due to the variation of T, in order to evaluate correctly the curves, we need to normalize the time and therefore the FoM. Figure 12 shows the results of experiment proposed.

Although the minimum charging current follows a quadratic function, in order to simplify its usability, we suppose that it follows a linear function if we assume an ±5% of error. This is the case in Figure 12. As we expected, the higher the iteration time *T* becomes for a given duty cycle, the greater the energy saving. In addition, the more the duty cycle *D* decreases, the more energy saving is increased. The Pareto front curve of the minimum required charging current increases its values in the same degree that the duty cycle decreases.

### 6.2. Optimal Design Curve Usage

**Figure 13:**
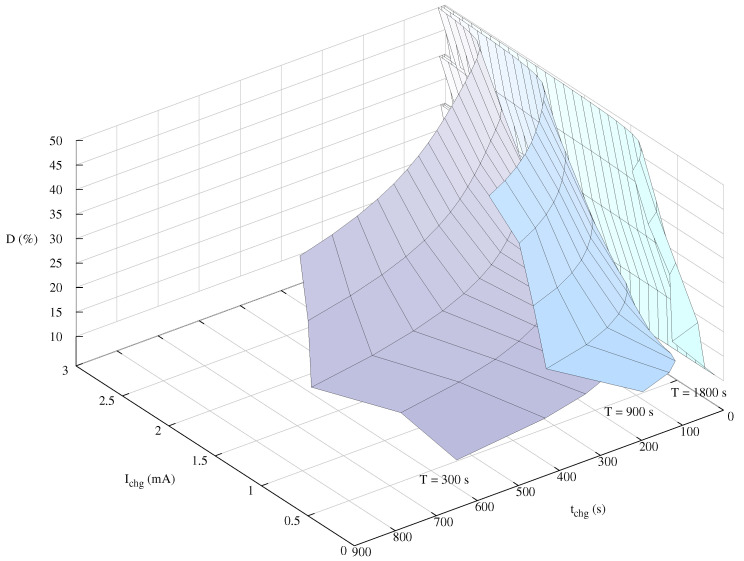
Optimal design space curves for different iteration times.

Figure 13 presents the complete optimal design curve of charging times, currents, and duty cycle for iteration times *T* equal to 1800, 900 and 300 s.

In this Figure 13, the light green curve represents the minimum optimal charging current and time for a given duty cycle when the iteration time of the sensor node is 1800 s, i.e., 30 min. The blue curve shows the same behavior in case of a duty cycle of 15 min (900 s). Finally, the purple surface shows the optimal values for a 300 s iteration time (*T* = 5 min).

These design curves help the designers to determine the optimal working modes of the underwater sensor node charger. For instance, in case the parasitic resistance of the energy transmission antenna is high, since the section of its cables’ cross section cannot be increased, the designers may determine with the help of these curves the optimal charging currents and times.

However, in order to understand the advantages of using our design exploration methodololy to optimize the energy transmision from the power source point of view, we propose to compare our results with other methodologies in the same scenario. As it was pointed out in Section 1, the related literature provides two solutions. The first and most extended approach consists in setting the sensor network energy provider to a constant powering. We call this first methodology always-on. The second approach presented in the literature continues using the worst case energy requirement but limits the powering in time. In this sense, the powering to the sensor network is activated just to fulfill the energy requirements from the execution of the measurement (see $t_{chg}$ in Figure 5). We call this second methodology timed-on. In both cases, the designers use the worst case energy requirement from the sensor network specifications.

In our case, as we are exploring the design space, we are able to adapt the energy supplied to the energy demanded instead of applying the worst case in all the scenarios. The used sensor node consumes 3 mA during the execution of a turbidity measurement. In addition, it is also necessary to translate this energy requirement to the power supply specifications. If we use a sensor network transmission system like the one presented in [14], the WPT through the sensor network produces an attenuation of 20 dB. In case of using the proposal presented in [15], the network attenuation is 15.55 dB in a 5-level sensor network. Therefore, the real current in the worst case at the power source is 28.8 mA and 17.97 mA, respectively.

Those consumption values are related to the execution charging time of the sensor node. Otherwise, in the best case, the sensor node is not consuming energy from its sensor network. However, we need to take into consideration that those approaches promote the WPT transmission distance using magnetic fields from 10 to 30 m. Certainly, the cost of extending the distance is that the power consumption of the sensor node is practically negligible in comparison with the WPT architecture deployed in the sensor network, e.g., receiving 1 W from an emission of 100 W as in [14]. In order to simplify the comparison, we assume that the sensor network does not have any power consumption. If a designer wants to take into consideration this attenuation, the solution just consists of adding to the sensor node the required energy for the attenuated power due to the usage of the sensor network.

Other open question is to understand the usage of the FoM and *D* functions (see Equations (14) and (15) for more details). A strict mathematical analysis of the FoM function indicates that it only compares the timed-on with the always-on methodologies. Please remember that those methodologies do not modify the charging current ($I_{chg}$). Therefore, the value to the right (3 mA) of Figure 10 shows this comparison. For example, given a 10% duty cycle, the timed-on methodology decreases the energy consumption 90% against the always-on methodology.

Including the $I_{chg}$ dependency is mandatory to evaluate our proposal. Then, this Figure 11 representing $FoM(I_{chg},D)$ is the comparison function between a design solution selected from our obtained TC and the always-on method. Note that the worst case of our proposal implies obtaining the timed-on methodology. In other words, we need to use the maximum charging current using the specified duty cycle *D*.

At this point, a new question arises: how we can compare our proposal with the timed-on methodology. Since the value to the right in each curve is the maximum $I_{chg}$ and it is used by the other two approaches in the literature, the horizontal distance between this maximum value and that required by our solution is just the advantage that we are looking for. As an example, given a 10% duty cycle and selecting a solution from the TC which requires $I_{chg}^{\mathrm{TC}}$=2 mA, the advantage can be computed as:

(18) $$distance\left(mA\right)=\max\left(I_{chg}\right)-I_{chg}^{\mathrm{TC}}=3-2=1\;\mathrm{mA}$$

(19) $$distance\left(dB\right)=20\times \log\frac{I_{chg}^{\mathrm{TC}}}{max(I_{chg})}=-3.52\;\mathrm{dB}$$

Therefore, we have reduced the instantaneous current 1 mA which represents half of the required power when a timed-on methodology is applied. In addition, the next question is how much we can reduce the power consumption without losing the timed-on over the always-on advantage. The response is: up to the Pareto front $PF(I_{chg}^{*},D)$ (see Equation (17)). Crossing this frontier would mean that the system is not using the high rate charging currents.

In other words, the sensor node is consuming more energy than that provided by the power supply. Therefore, the sensor network charges the sensor node battery or energy accumulator to compensate the required energy to the provided one, and this operation requires more charging time than the high rate charging currents. This is the reason why the values to the left of this $PF(I_{chg}^{*},D)$ drop the FoM.

Although those left design points from the $PF(I_{chg}^{*},D)$ limit reduce the FoM, their existence demonstrates that the studied sensor network is able to perform experiments in their sensor nodes, which requires more energy than the maximum instantaneous energy provided by the sensor node power source.

Table 2 summarizes all the critical design points explained for the experiment presented in Figure 11. The last column of this table presents the advantage in terms of provided power in comparison with the timed-on methodology keeping the FoM close to a 5% of the max(FoM) as exposed in Equation (17). We can conclude that our approach requires in the worst case half the power compared to the best approach found in the literature. Moreover, this advantage increases up to 17.95 dB for the best case. This advantage is 62.37 times less power than the best approach referred.

### 6.3. Computational Effort Comparisons

In this section, we evaluate the computational effort of our approach. We must take into consideration that our exploration methodology is based on a double loop algorithm. We are concerned that the main computational effort is caused by the inner loop. Computational effort of the external one is limited to applying the practical considerations exposed throughout this paper. Therefore, we focus this study on evaluating the inner loop computational effort.

Since each individual evaluation of our NSGA-II application requires a Simulink circuit simulation of the underwater sensor node and its battery models, the cost of the other optimization engine procedures is negligible. Each simulation requires about 5.2 s to be executed on an Intel i7-4930K microprocessor with 64 GB RAM using a single core at 3.4 GHz running Windows 10 Pro, Matlab v9.10, and Simulink v10.3.

Based on the conclusions from experiments in Section 4.3 concerning Convergence, we obtained each point of the curve presented in Figure 13 using a 10-individual population in 14 generations, what has meant to perform 140 simulations for obtaining each point. The curve for an iteration time *T* equal to 30 min is composed of 277 points.

In order to make comparisons with other methodologies, let us first assume the accuracy reached with our approach as we made previously in Section 4.5:

(20) $$I_{chg}\times t_{chg}<0.1\;\mu \mathrm{Ah}$$

In addition, we assume a target battery voltage in the range $V_{chg}\in \left[2.65,2.75\right]$ V, the charging current in the range $I_{chg}\in \left[0.2,2.2\right]$ mA and the charging time $t_{chg}\in \left[300,600\right]$ s. For those values, the error may be rewritten as:

(21) $$\left(\max\left(I_{chg}\right)-\min\left(I_{chg}\right)\right)\times \left(\max\left(t_{chg}\right)-\min\left(t_{chg}\right)\right)\times \delta <0.1\;\mu \mathrm{Ah}$$

(22) $$2\times 10^{-3}\times 300\times \delta <0.1\times 10^{-6}$$

(23) $$\delta <1.67\times 10^{-7}$$

where δ is the accuracy coefficient. In other words, the minimum variation of the product of the $I_{chg}$ and $t_{chg}$ design variables must be at least δ. An exhaustive search algorithm based on a double loop, one per design variable, is not practical. Nevertheless, we can use a bisection approach to reduce dramatically the big amount of points that would result from an exhaustive double loop approach. The required *N* iterations for a one design variable dimension search approach based on bisection can be computed as:

(24) $$N\geq 24>1-\frac{\log(\delta )}{\log(2)}=23.52$$

Since the bisection search should be executed on a two-dimensional space, it requires 576 simulations. This represents four times the number of simulations in comparison with the NSGA-II approach presented in this paper. We need to mention that this computational effort evaluation represents the best case scenario of a bisection search approach. This percentage is greater when the charging current and time ranges increase and the duty cycle is reduced. For instance, if $t_{chg}\in \left[300,1800\right]$ and *D* = 10%, with the same current range, the advantage of our proposed approach is 4.8 times faster than the bisection search strategy.

## 7. Conclusions

In this paper, the design exploration of the charging current–time tradeoff curve of a practical underwater wireless sensor node has been studied in detail. After modeling the sensor node and its battery, our local search strategy based on NSGA-II is presented. The problem codification and fitness function are studied based on practical considerations. The quality of the proposed approach is evaluated through its convergence. Exploring a local set of optimal design points of the search space, the local Pareto fronts of those solutions are obtained based on the charged voltage in the battery. An additional figure of merit is proposed and evaluated during several experiments to determine the design Pareto fronts. From the discussion, we determine the high/low charging current frontier and also the minimum charging current frontier. Using those curves, the complete tradeoff curve of charging current and time and also working duty cycle is obtained. Our heuristic proposal is compared in terms of computational effort, measured in number of circuits simulations, with a bisection search approach. Finally, from the comparisons, we conclude that, given an accuracy of 0.1 μAh for the charging current–time product, our approach is at least four times better than the bisection approach in terms of computational effort. In terms of power supplied, our approach reduces the power consumption at least 3.3 dB and 17.95 dB in the worst and the best cases tested, respectively.

## Figures and Tables

**Table 1:** Simulation parameters for the underwater sensor node battery.

Parameter	Value
Model	UMAC040130A003TA01
Target Application	Backup Battery
Nominal Capacity	3.0 mAh
Nominal Voltage	2.3 V
Capacity at Nominal Voltage	2.31 mAh
Response Time	30 s
Maximum Capacity	3.06 Ah
Exponential Zone Voltage	2.46 V
Exponential Zone Capacity	0.12 mAh
Nominal Discharge Current	3.0 mA
Cut-off Voltage	1.8 V
Full Charged Voltage	2.7 V
Internal Resistance	0.8 Ω

**Table 2:** Critical design points from the Pareto front for minimum charging current and high/low charging currents.

*D*	max(FoM) ${}^{1}$	$\mathrm{FoM}(I_{\mathrm{chg}}^{*},D)$	$I_{\mathrm{chg}}^{*}$	$\mathrm{FoM}(\min\left(I_{\mathrm{chg}}\right),D)$	$\min(I_{\mathrm{chg}})$	(Distance)
(%)	(%)	(%)	(mA)	(%)	(mA)	(dB)
10	89.80	85.99	0.4	63.41	0.2	−17.95
20	79.65	75.30	0.9	39.56	0.3	−10.95
30	69.10	65.65	1.3	31.05	0.5	−7.47
40	59.38	56.80	1.7	22.27	0.7	−5.19
50	49.08	47.70	2.0	6.55	0.8	−3.31

${}^{1}$ $max(I_{chg})$ = 1mA, *T* = 900 s.

## Data Availability

Data sharing not applicable.
